# Comparison of Decellularization Protocols to Generate Peripheral Nerve Grafts: A Study on Rat Sciatic Nerves

**DOI:** 10.3390/ijms22052389

**Published:** 2021-02-27

**Authors:** Marwa El Soury, Óscar Darío García-García, Matteo Moretti, Isabelle Perroteau, Stefania Raimondo, Arianna Barbara Lovati, Víctor Carriel

**Affiliations:** 1 Department of Clinical and Biological Sciences, University of Torino, 10043 Orbassano, Italy; marwa.elsoury@unito.it (M.E.S.); isabelle.perroteau@unito.it (I.P.); 2 Neuroscience Institute Cavalieri Ottolenghi (NICO), University of Torino, 10043 Orbassano, Italy; 3 Tissue Engineering Group, Department of Histology, University of Granada, 18012 Granada, Spain; e.oscargg@go.ugr.es (Ó.D.G.-G.); vcarriel@ugr.es (V.C.); 4 Instituto de Investigacion Biosanitaria, Ibs.GRANADA, 18012 Granada, Spain; 5 IRCCS Istituto Ortopedico Galeazzi, Cell and Tissue Engineering Laboratory, 20161 Milan, Italy; matteo.moretti@grupposandonato.it (M.M.); arianna.lovati@grupposandonato.it (A.B.L.); 6 Regenerative Medicine Technologies Laboratory, Ente Ospedaliero Cantonale, 6900 Lugano, Switzerland

**Keywords:** peripheral nerves, decellularization, acellular, regeneration, orthopedic trauma, extracellular matrix

## Abstract

In critical nerve gap repair, decellularized nerve allografts are considered a promising tissue engineering strategy that can provide superior regeneration results compared to nerve conduits. Decellularized nerves offer a well-conserved extracellular matrix component that has proven to play an important role in supporting axonal guiding and peripheral nerve regeneration. Up to now, the known decellularized techniques are time and effort consuming. The present study, performed on rat sciatic nerves, aims at investigating a novel nerve decellularization protocol able to combine an effective decellularization in short time with a good preservation of the extracellular matrix component. To do this, a decellularization protocol proven to be efficient for tendons (DN-P1) was compared with a decellularization protocol specifically developed for nerves (DN-P2). The outcomes of both the decellularization protocols were assessed by a series of in vitro evaluations, including qualitative and quantitative histological and immunohistochemical analyses, DNA quantification, SEM and TEM ultrastructural analyses, mechanical testing, and viability assay. The overall results showed that DN-P1 could provide promising results if tested in vivo, as the in vitro characterization demonstrated that DN-P1 conserved a better ultrastructure and ECM components compared to DN-P2. Most importantly, DN-P1 was shown to be highly biocompatible, supporting a greater number of viable metabolically active cells.

## 1. Introduction

Despite the known innate capacity of peripheral nerves to regenerate injuries, in most of the cases, the desired complete functional recovery is seldom achieved. Numerous factors govern the success of nerve regeneration, of which the severity of the injury plays a key role. In 1947, Seddon classified peripheral nerve injuries into three grades of severity: neuropraxia, axonotmesis, and neurotmesis. While the first two types of injury can regenerate spontaneously, in neurotmesis, surgical intervention is usually required [1]. In 1951, a more detailed and accurate nerve injury classification was made by Sunderland, where Type I corresponds to neuropraxia, Types II, III, and IV are equivalent to axonotmesis, and Types V and VI correspond to neurotmesis and mixed grades of nerve injury [2]. In the case of neurotmesis injuries, where the nerve continuity is completely disrupted, the repairing strategy is adopted depending on whether the injury is accompanied by nerve substance loss or not.

In simple nerve transections unaccompanied by substance loss, tensionless direct surgical neurorrhaphy is the optimal choice. When tensionless epineural neurorrhaphy is impossible, nerve scaffolds must be used to join the transected nerve stumps [3]. In this case, autografts are considered the gold standard technique. Unfortunately, despite providing optimal results concerning nerve regeneration and subsequent functional recovery, this technique is associated with numerous drawbacks, i.e., limited availability, two-step surgery, loss of sensitivity, donor site morbidity, and neuroma formation at the donor nerve site [4]. 

To overcome this problem, attention was drawn to find other alternatives to join the two distant nerve stumps. With this scope and the aid of tissue engineering techniques, various nerve scaffolds were obtained, including nerve guidance conduits of both natural and synthetic origins. Non-nervous tissues, including tendon, vein, artery, and muscular scaffolds, were also used for nerve repair, as well as allografts [5]. Providing nerve allograft from cadaver donors was thought to be a suitable alternative to autografts, but concurrent immunosuppressive treatments are required to prevent adverse immune reactions and subsequent graft rejection [6]. Eliminating the cellular antigens while conserving the nerve extracellular matrix (ECM) and its structure was the main goal behind decellularization techniques.

Tissue decellularization can be achieved by combining physical factors with different chemical and biological factors. Various physical agents such as tissue exposure to thermal change, agitation, and ultrasonic waves can help in the process of decellularization. Changing temperatures through repeated cycles of freezing and thawing can effectively lyse the cells, but the membranous and cellular remains need subsequent processing for an effective removal. Hence, the combination of different chemical, biological, and physical factors is commonly required [7,8,9,10].

Chemical factors include the use of acidic and basic solutions that catalyze the hydrolytic degradation of biomolecules. Hypo- and hypertonic solutions cause cell lysis by changing the cell osmotic pressure. Detergents including ionic, non-ionic, and zwitterionic ones solubilize cell membranes and remove cellular material from tissues. Organic solvents are also used, such as tributyl phosphate (TBP), that were demonstrated to be more effective in decellularizing dense tissues as tendons compared to other detergents like Triton-X100 and sodium dodecyl sulfate (SDS) [11]. Biological agents mainly include enzymes (nucleases, chondroitinase ABC, trypsin, collagenase); in particular, DNase and RNase are used to complete the removal of nucleic acid remained in the tissue. However, an insufficient removal of chemical detergents from the processed tissue can result in high cytotoxicity, as also reported elsewhere [12]. Therefore, extensive washing of chemical detergents should be considered, or other solutions can be employed, such as peracetic acid. Indeed, Peracetic acid (PAA) has been commonly reported as a potent oxidizing agent used to sterilize collagen tissues [13] while enhancing the tissue permeability for the detergent penetration [14,15,16,17,18,19]. Thanks to the PAA activity, the total amount and concentration of detergents could be drastically reduced [12], thus also decreasing the presence of remains in the tissues and therefore their potential toxicity.

Decellularized nerves, also known as acellular nerve grafts, provide the adequate preservation of the internal nerve structure where endoneurial tubes, basal lamina, and laminin remain intact, thus facilitating the process of axonal regeneration. Certainly, during the decellularization process, some alterations of the ECM composition and some ultrastructure disruptions would be unavoidable, but a good decellularization method would minimize these undesirable effects. Up until now, three main decellularization protocols are described in the creation of a functional nerve graft: (i) the one described by Sondell and colleagues (Triton X-100 and sodium deoxycholate “SDC”) [9]; (ii) the protocol described by Hudson and colleagues (Sulfobetaine _SB-10 and SB-16) [8], the only one patented and available in US market as Avance^®^ Nerve Graft (AxoGen Inc. Alachua, FL, USA); and (iii) the combined Hudson protocol added with chondroitinase ABC by Krekoski and colleagues [20]. However, all these decellularization methods are very long and time-consuming, giving rise to risks of contamination and technical unsuitability [21]. A more recent strategy to perform the nerve decellularization has been proposed by Boriani and colleagues (SB-10, TritonX-100, and SDS) [22] with the advantage of speeding up myelin and cellular debris detachment, without detrimental effects on the nerve architecture, and without breaking the aseptic chain.

The aim of the present study is to test a nerve decellularization method that could be less time consuming and require less reagents to minimize the exposure to detergents [23]. Moreover, it would be able to combine an effective decellularization in short time with a good preservation of the ECM [23]. The investigated protocol combined the use of two main reagents: TBP—to our knowledge firstly described to decellularize nerves—and PAA. TBP was hypothesized to be able to penetrate the compact nerve structure better than other detergents. With the aim to enhance the detergent penetration and to achieve the sterilization of the decellularized nerves, the use of the PAA has been planned too. Although the use of the PAA showed very promising outcomes in several tissue decellularization processes [21], its efficacy still needs to be further analyzed for the nerve decellularization, since it has been barely described in the literature as a disinfectant agent [24,25]. The PAA was previously tested to decellularize tendon xenografts, demonstrating a good efficacy [11]. Since both nerves and tendons are scarcely permeable, it was hypothesized that the use of this promising decellularization protocol for nerves could efficiently produce biocompatible and well-structured acellular nerve allografts. 

To evaluate and compare the efficacy of our protocol to decellularize peripheral nerves, another previously published decellularization protocol specifically developed for this tissue [22] has been used as control.

## 2. Results

### 2.1. Evaluation of Nerve General Structure and ECM Components

**Figure 1:**
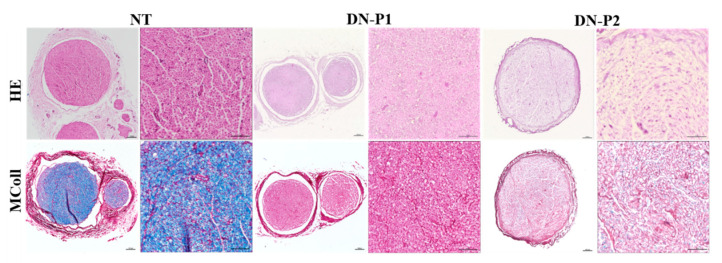
Representative panel of native and decellularized rat sciatic nerves reporting the morphological architecture. Sections stained with hematoxylin and eosin (HE) identify the overall nerve histoarchitecture. MCOLL staining shows myelin (blue) and collagen (red) simultaneously. Scale bar = 100 µm for lower magnification images and 50 µm for higher magnification ones.

The main aim of the decellularization process is to obtain non-immunogenic nerve scaffolds of natural origin by removing cells and antigens while preserving the functional structure of the tissue. Nerves were histologically and immunohistochemically stained to determine changes in structure and cellular components compared to intact native nerves used as controls. Hematoxylin and eosin staining showed a good conservation of overall nerve structure with DN-P1; in particular, the endoneurium and perineurium appeared well-conserved with visible cylindrical structures. Differently, DN-P2 resulted in a moderate disruption of the endoneurium and perineurium (Figure 1). 

At higher magnifications, MCOLL histochemical method simultaneously stained the myelin and fibrillar collagens and detected a few myelin remnants in nerves treated with DN-P2 but not in those treated with DN-P1 that showed a complete myelin removal (at light microscopy). Collagen was intensely stained in DN-P1, while this histochemical reaction was weaker in DN-P2 (Figure 1). 

Immunohistochemical staining of S100 (Schwann cells) and vimentin (fibroblasts) detected a greater presence of Schwann cell and fibroblast remnants in DN-P1 than in DN-P2 group. The semiquantitative analyses of S100 confirmed the removal of these elements when compared to native nerves; DN-P1 was statistically lower compared to natives and DN-P2 statistically significant compared to both natives and DN-P1 samples. Vimentin semiquantitative analysis did not show significant differences between DN-P1 and native samples, while DN-P2 samples showed a significant vimentin decrease compared to native and DN-P1 nerves.

**Figure 2:**
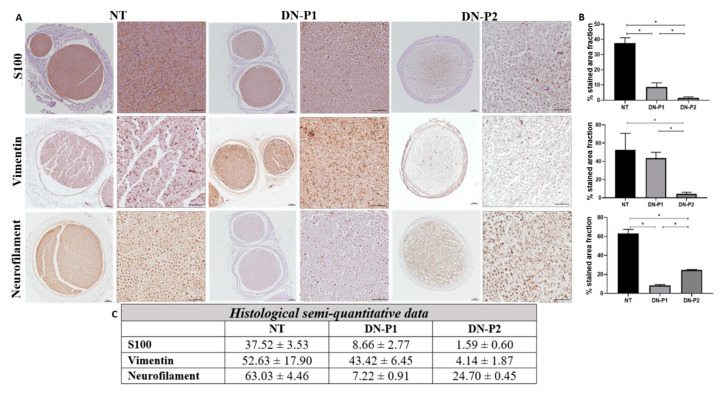
Representative immunohistochemical images and semiquantitative results of native and decellularized rat sciatic nerves reporting different cellular components. (**A**) The immunostaining by S100, Vimentin, and Neurofilament show the Schwann cells, fibroblasts, and axons, respectively. Scale bar = 100 µm for lower magnification images and 50 µm for higher magnification images. (**B**) Graphs representing semiquantitative analyses of the % occupied by each immunoreaction normalized to the whole nerve area. (**C**) Semi-quantitative raw data. * indicate statistically significant differences (*p* < 0.05).

However, immunohistochemical staining for neurofilament (axons) showed a better removal of these neuronal proteins in nerves treated with DN-P1 compared to those treated with DN-P2 (Figure 2A). This data was confirmed also by the semiquantitative analysis where DN-P1 was significantly less compared to native and DN-P2 nerves (Figure 2B,C).

**Figure 3:**
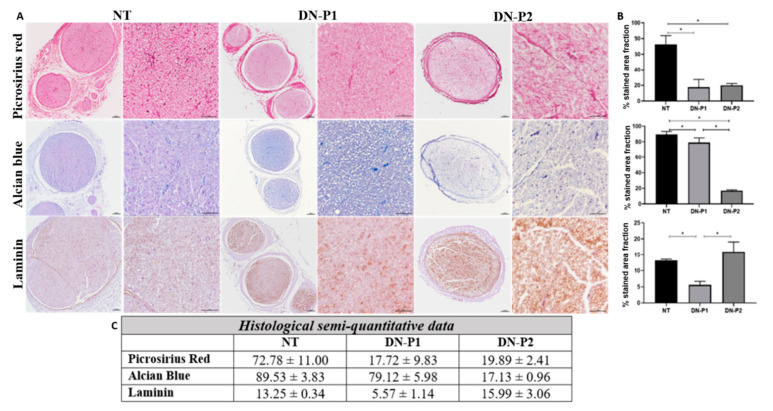
Representative histological and immunohistochemical panels and semiquantitative analysis of native and decellularized rat sciatic nerves reporting the ECM components. (**A**) The three main ECM components, i.e., collagen, proteoglycans, and laminin, are stained with Picrosirius red, Alcian blue, and laminin immunostaining, respectively. Scale bar = 100 µm for lower magnification images and 50 µm for higher magnification images. (**B**) Graphs representing semiquantitative analysis of the % occupied by the staining normalized to the whole nerve area. (**C**) Semi-quantitative raw data. * indicate statistically significant differences (*p* < 0.05).

Conservation of the ECM in decellularized nerve is crucial, as they sustain the Schwann cell migration and axonal re-growth. Nerves were histochemically stained with Picrosirius red and Alcian blue to qualitatively evaluate the presence of collagen and acid proteoglycans, respectively (Figure 3A). The semiquantitative analyses revealed that nerves treated with both protocols resulted in a significant reduction of the histochemical reaction against collagens as compared to native nerves (*p* < 0.05). No significant differences were observed between both the experimental conditions (*p* = 0.513) (Figure 3B,C). The analysis of the proteoglycans confirmed a clear removal of these non-fibrillar molecules from the epi-, peri-, and endoneurium levels with both the decellularization protocols as compared to native controls (*p* < 0.05). This reduction was more evident and significant in nerves treated with DN-P2 compared to those treated with DN-P1 (*p* < 0.05). Furthermore, the basal membrane glycoprotein laminin was also immunohistochemically identified due to its critical role during regeneration. These results revealed a better preservation of these molecules in nerves treated with DN-P2 compared to DN-P1 treated nerves. However, the organization pattern and distribution, obtained after decellularization, differed with respect to the well-delimited basal membranes observed in native nerves (Figure 3A). Laminin quantitative analyses confirmed the significant reduction of the molecule in nerves treated with DN-P1 compared to native and DN-P2 nerves (*p* < 0.05). Moreover, no statistical differences were observed in nerves treated with DN-P2 compared to the native condition (*p* = 0.513) (Figure 3B,C).

### 2.2. DNA Detection 

**Figure 4:**
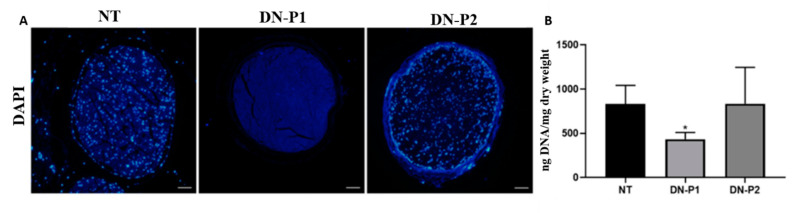
Qualitative and quantitative DNA content of native and decellularized rat sciatic nerves evaluating the effectiveness of the decellularization process to remove cell component. (**A**) Sections labeled with DAPI identify the cell nuclei (blue); scale bar =100 µm. (**B**) DNA quantification shows a lower DNA content in nerves decellularized with DN-P1 compared to both native tissue and nerves decellularized with DN-P2; * indicate statistically significant differences (*p* < 0.05).

Concerning DNA testing, both fluorescent DAPI staining and DNA quantification were performed (Figure 4). Images did not show DAPI staining for DNA remnants in DN-P1, while several cell nuclei were observed in DN-P2 samples (Figure 4A). These findings were confirmed by the quantitative spectrophotometric evaluation of purified DNA. In fact, a halved DNA content was observed in nerves decellularized with DN-P1 (432.98 ± 62.02 ng DNA/mg dry weight) compared to specimens treated with DN-P2 (834.83 ± 335.49 ng DNA/mg dry weight) or native tissue (831.87 ± 171.37 ng DNA/mg dry weight) (Figure 4B). 

### 2.3. Ultrastructural Changes in Decellularized Nerves: Scanning and Transmission Electron Microscopy

**Figure 5:**
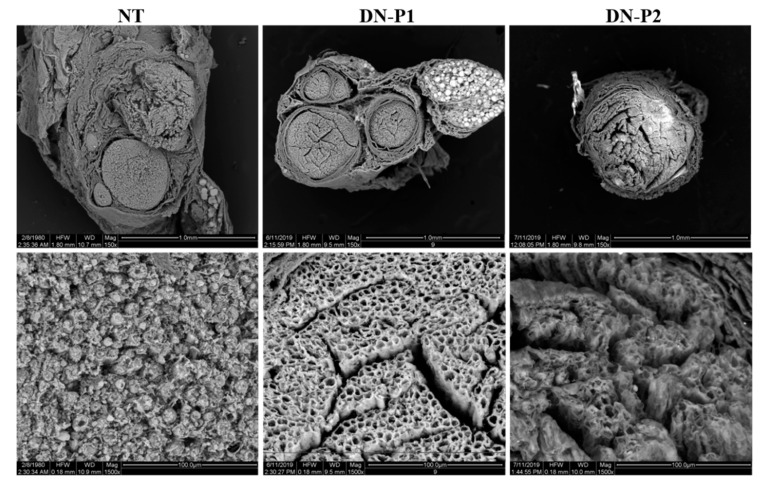
Scanning electron microscopy (SEM) representative panel. SEM detects the surface ultrastructure and the changes occurred in rat sciatic nerves following the decellularization process.

Electron microscopy of the decellularized nerves was performed to detect changes at the nerve ultrastructural level. Nerve samples were analyzed using both scanning (SEM) and transmission electron microscope (TEM) to detect the nerve surface and inner ultrastructure, respectively. SEM analysis demonstrated a very well-preserved ultrastructure of nerves treated with DN-P1 (Figure 5). Indeed, the nerves endoneurial tubes showed a well-organized net-like structure of the ECM in which a well-perforated surface can be seen. Instead, nerves treated with DN-P2 showed a less organized structure, and the net-shaped structure was not well detected. In particular, the ECM appeared severely disrupted. In native nerves, conserved nerve fascicles were easily detectable.

**Figure 6:**
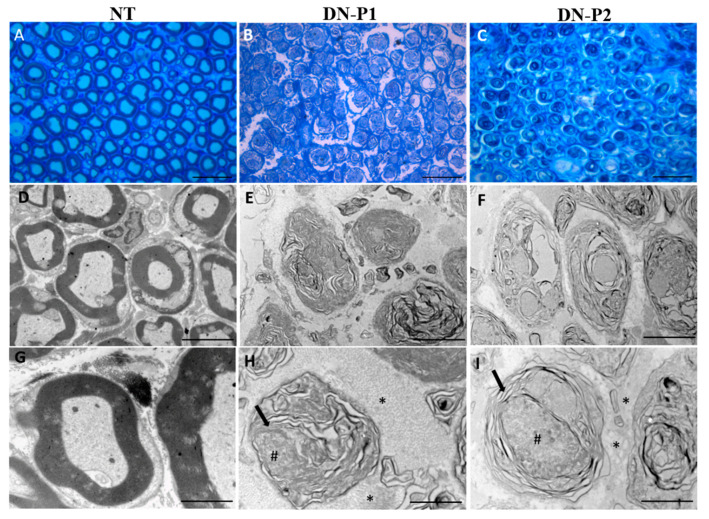
Toluidine blue staining and Ttansmission electron microscopy (TEM) representative panel. Toluidine blue stained sections (**A**–**C**) show the overall structure of the decellularized rat sciatic nerves. TEM (**D**–**I**) detects the changes in the inner ultrastructure of the native tissue and decellularized nerves. Myelin structures (black arrow), collagen fibers (*), axon remnants (#). (**A**–**C**) scale bar = 20 µm; (**D**–**F**) scale bar = 5 µm; (**G**–**I**) scale bar = 2 µm.

Concerning the inner ultrastructure of the decellularized nerves, TEM analysis (Figure 6) showed that unfolded myelin structures were still present in nerves treated with both the decellularization protocols. TEM analyses accurately demonstrated that a complete myelin removal was not achieved in any of the tested samples; actually, multilayered cellular structures with low lipid content were identified (Figure 6). Structurally altered axonal remnants were detected in DN-P1, while in DN-P2 treated samples, some relatively intact axonal structures were still present. Collagen-rich ECM could be detected in samples treated with both protocols, even though well-conserved collagen fibers were detected only in samples treated with DN-P1. Otherwise, misfolded unintegrated collagen fibers were seen in DN-P2 specimens.

### 2.4. Biomechanical Properties

**Figure 7:**
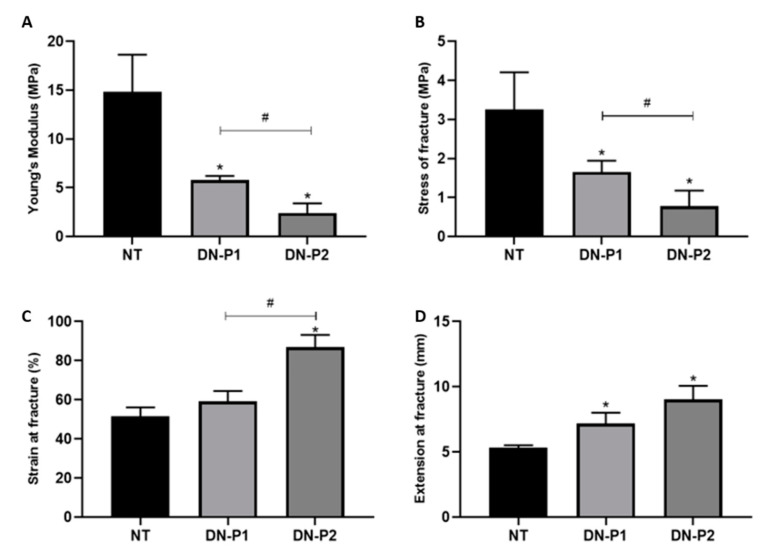
Biomechanical properties of native and decellularized rat sciatic nerves. (**A**) Young’s modulus (MPa), (**B**) failure stress (MPa), (**C**) failure strain (%), and (**D**) elastic modulus (mm). * indicate statistically significant differences (*p* < 0.05) of decellularization protocols compared to natives, # indicate statistically significant differences (*p* ˂ 0.05) between the two decellularization protocols.

Biomechanical properties of the samples were measured to determine potential changes induced by detergents and by the entire decellularization process. Both the decellularization protocols induced a low stiffness measured by the Young’s modulus. Specifically, native nerves measured 14.82 ± 3.84 MPa, while DN-P1 and 2 registered 5.80 ± 0.43 and 2.41 ± 1 MPa, respectively (Figure 7A). Consequently, failure stress showed a similar behavior in native nerves 3.26 ± 0.95 MPa and samples treated with DN-P1 and 2 for 1.65 ± 0.29 and 0.78 ± 0.40 MPa, respectively (Figure 7B). Concerning the deformation that occurred in nerves at the point of failure, the strain of nerves treated with DN-P1 was slightly higher than that of native nerves despite not being significant, measuring 59.32 ± 5.19 and 51.50 ± 4.66 MPa, respectively. Nerves treated with DN-P2 had higher strain values (86.84 ± 6.37 MPa) compared to both native controls and nerves treated with DN-P1 (Figure 7C). The strain at fracture was significantly higher in both the decellularized groups (7.19 ± 0.83 and 9.03 ± 1.05 MPa for DN-P1 and 2, respectively) compared to native controls 5.34 ± 0.18 MPa (Figure 7D).

### 2.5. Qualitative Data of Cell Viability

**Figure 8:**
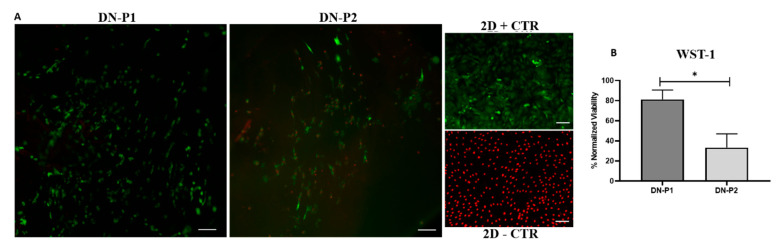
Representative qualitative and quantitative panel of Live & Dead and WST-1 assay of ADMSC-reseeded decellularized rat sciatic nerves. (**A**) Live & Dead (L/D) analysis. Green cells are alive while the red ones are dead. Scale bar 50 µm. (**B**) WST-1 analysis, normalized viability to 2D + CTR expressed in percentage. * indicate statistically significant differences between the two decellularization protocols (*p* < 0.05).

The Live & Dead assay showed attached viable cells seeded on the inner surface of decellularized nerves following 48 h of standard culture conditions. As can be seen in 2D positive and negative controls, cells are detected in green or red colors, respectively. More importantly, a greater number of viable and elongated cells were detected within the nerves treated with DN-P1, while viable cells were less abundant in DN-P2 group. In relation to the presence of dead cells, they were not detected in nerves decellularized with DN-P1. However, the specimens decellularized with DN-P2 showed several dead cells on their surface (Figure 8A). These results were confirmed by WST-1 biochemical assay, where DN-P1 showed a comparable cellular metabolic activity with 2D positive controls, metabolic activity was slightly lower compared to 2D positive controls, and statistical analysis was not significant (*p* = 0.121) (Figure 8B). DN-P1 showed much higher metabolic activity compared to 2D negative control (*p* = 0.0431). DN-P1 showed higher WST-1 values and therefore a greater cellular metabolic activity compared to DN-P2, these differences being statistically significant (*p* = 0.046). DN-P2 metabolic activity was significantly lower when compared to 2D positive controls (*p* = 0.049) and DN-P1 (*p* = 0.046) but was significantly higher when compared to 2D negative control (*p* = 0.046) (Figure 8B). Cells seeded on DN-P1 demonstrated a mean value of 82% viability (81 ± 9. 38), while DN-P2 demonstrated a mean value of 33% viability (33.39 ± 13.63). Difference between the two groups was significant (*p* = 0.046).

## 3. Discussion

In the search for other valid alternatives to substitute the gold standard repairing technique (autograft), different tissue engineered nerve conduits have been developed. Unfortunately, nerve conduit efficiency is limited just to repairing small nerve gaps up to 3 cm [26]. This phenomenon is likely to be attributed to the absence of the cellular and ECM components that have been readily supported by the autograft to the injured nerve. The enrichment of hollow conduits with cells and ECM-derived hydrogels has been demonstrated to augment the regeneration, but its clinical application is limited, being both time and effort consuming. Decellularized peripheral nerve allografts could be a more promising alternative in repairing critical nerve gaps when compared to hollow conduits. 

Decellularized nerve allografts would provide a nerve scaffold rich in ECM components with an internal organized 3D structure formed by aligned endoneurial tubes. Furthermore, decellularized nerves offer a natural and tissue-specific mechanical support to the regenerative microenvironment. Indeed, the presence of well-distributed essential ECM, such as laminin and/or collagens, acts as guidance cues for Schwann cell migration and subsequent axon regrowth [27], decreasing or avoiding the host immunological response [28]. Therefore, the preservation of the ECM 3D organization and molecular composition is an important condition after the nerve decellularization [23].

In this study, we aimed at evaluating the effectiveness of an already developed decellularization protocol for tendons [11], both in terms of cellular removal and ECM structural and molecular preservation when applied on rat sciatic nerves. To demonstrate the efficacy of DN-P1, here we compared this procedure with another detergent-based decellularization protocol specifically developed for nerves by others [22]. With this aim, in vitro comprehensive analyses were performed including histology (HE), histochemistry (Picrosirius red, Alcian blue, DAPI, and MCOLL), immunohistochemistry (S100, vimentin, neurofilament, and laminin), DNA quantification, SEM and TEM analyses, and biomechanical testing. In addition, the biological properties and cytocompatibility were determined through the use of cell viability and functionality assays (L/D and WST-1). In particular, the objective of the present study was to assess if detergents frequently used to decellularize dense connective tissues as tendons (TBP, PAA, and DNase) could better preserve the ECM components of the treated nerves compared to Triton-X100 and SDS. The latter reagents are considered to be very aggressive decellularizing agents. Moreover, the development of a quick, efficacious protocol to decellularize nerves is strongly required to reduce the risk of contaminations during the process and to respond to clean room productions. 

Overall, we demonstrated that the two applied decellularization protocols (DN-P1 and DN-P2) convey different conservation levels of the main ECM components: collagen, proteoglycans, and laminin. Both protocols decreased the collagen content almost to the same extent, while DN-P1 better preserved proteoglycans and DN-P2 had a higher presence of laminin, but more disorganized. Immunohistochemically, our results showed that DN-P1 and DN-P2 had alternative points of strengths and weaknesses; for example, DN-P1 had a better nerve structural preservation, while DN-P2 showed a better removal of the cellular components. These findings were not confirmed by DAPI and DNA content that demonstrated a greater cellular removal in samples treated with DN-P1 compared to DN-P2. Therefore, these results point out the usefulness of the immunohistochemistry to accurately confirm the removal of tissue-specific cellular components after decellularization [23]. MCOLL histochemistry for myelin and collagen showed that DN-P1 induced a better myelin removal and collagen preservation than DN-P2. In addition, SEM analysis greatly confirmed these findings, revealing a well-conserved ECM structure in DN-P1 rather than in DN-P2 samples. Finally, TEM ultrastructural evaluation was crucial to confirm and demonstrate the degree and efficacy of the decellularization protocols. Indeed, TEM has clearly confirmed the degree of conservation of the collagen matrix as well as the presence of axonal and cellular remnants, mainly those associated with the complex structure of the myelin sheath.

It needs to be taken into account that our study was performed on rat sciatic nerves, and this could explain the different results reached in the present study compared to the original protocol employed by Boriani and colleagues (DN-P2) that was applied on a diverse animal species, the rabbit [22]. These findings open an important and critical issue, i.e., the animal species as the source of nerve tissue. Indeed, there are substantial structural variations among nerves and among different animal species; the variations in nerve dimensions between different species can greatly affect the successfulness of the decellularization process. Unfortunately, these variations makes it difficult to compare different published studies in literature. As mentioned before [29] and as noticed in our results, each decellularization protocol should be adapted and optimized per each type of tissue, but also the species differences should be taken into account.

In any case, the best option for a clinical scenario would be the optimization of these decellularization methods to obtain human acellular nerve grafts from allogenic donors.

To our knowledge, just the studies that were published in collaboration with our group had widely compared in vitro rat sciatic decellularized nerves. The efficacy of different decellularization detergent-based protocols such as Sondell’s, Hudson’s, and the authors’ own protocol [30] originally developed for heart valves was tested [31]. Moreover, a recently published article comparing the effect of two different concentrations of genipin added as a natural crosslinking agent to the previously mentioned decellularization protocols of Sondell and Roosens was also tested [32] Indeed, in the present study, we found more similarities with the protocols tested on rat sciatic nerves proposed in these works [30,32] rather than with those developed by Boriani and colleagues on rabbit nerves [22]. However, further comparative studies are needed to determine the best option. In the case of the Roosens-based acellular nerve grafts [30], they were successfully used to repair 10-mm nerve gaps in rat, obtaining promising results, which were closely comparable to the efficacy of autograft technique [28].

It is well known that the immunogenicity of ECM of nerves is weak and almost negligible, indicating that cells are the main source of allogeneic nerve immunogenicity. The Major Histocompatibility Complex located on the surface of Schwann cells is the primary antigen material that induces an immune response against allograft transplantation. Therefore, cellular component removal is an important step in the preparation of decellularized nerve grafts [33]. 

A greater DNA removal was achieved using DN-P1, in which intact nuclei were completely absent, as demonstrated by DAPI staining; this was also found in the Roosens protocol alone or with the addition of genipin [30,32]. While the DNA quantification presented in Roosens protocol [30] achieved a less DNA content, on the other hand, the DNA quantification in DN-P1 and DN-P2 did not reach the limits considered optimal for decellularized tissues (50 ng/dry weight) [7], thus indicating that both protocols were not optimal to completely eliminate DNA, albeit a halved quantity was detected in DN-P1 compared to DN-P2. The decrease noted in DN-P1 compared to DN-P2 could be the effect of the additional DNase treatment in DN-P1, differently from DN-P2 that never applied a DNase treatment. 

It can be presumed that the absence of DAPI staining was due to an extreme fragmentation of the DNA that rendered it undetectable through this fluorochrome. DAPI is an intercalating fluorochrome that specifically recognizes the A–T interactions, and thus the breakage of DNA directly affects the DAPI staining. 

In the work presented by Bottagisio and colleagues [11] using DN-P1 to decellularize tendons, adequate DNA content < 50 ng/dry weight was achieved. The differences in the DNA content could be attributed to the fact that the decellularization protocol did not have the same efficiency on different tissues and different species, again highlighting the importance of optimizing the protocol based on these factors. To improve the DNA content removal in DN-P1, a greater concentration of DNase could be used; also, integrating the use of PAA in previous passages during the decellularization protocol could be helpful to obtain a better penetration of TBP and DNase. Augmenting the incubation times or slightly increasing the detergent concentrations could also be a possible strategy.

To our knowledge, the only decellularization protocol that was able to satisfy the recommended DNA content criteria is the Roosens’ protocol [30]. In that article, three decellularization protocols (Sondell, Hudson, and Roosens) were extensively tested in vitro. The DNA content revealed that Roosens demonstrated the least, followed by Sondell and then Hudson. Comparing our values with those presented in this article, we can assess that DN-P1 and DN-P2 demonstrated higher levels compared to both Roosens and Sondell, but lower compared to Hudson. The successful removal of DNA content observed in Roosens’ protocol could be attributed to a combined treatment of both DNase and RNase. While in the Sondell’s protocol, there were not any DNase incubation steps, we suggest that the stronger detergents used in that protocol could result in a better DNA component removal compared to our tested protocols.

Both histological and immunohistochemical analyses showed that ECM conservation was comparable between the two decellularization techniques, with almost the same extent of collagen amount. However, only DN-P1 showed a good conservation of collagen fibers. DN-P1 showed less removal of acid proteoglycans. DN-P1 also showed some levels of laminin conservation, though to a lower extent compared to DN-P2.

SEM and TEM analysis showed that DN-P1 demonstrated superior outcomes when compared to DN-P2. SEM showed a higher preservation of the endoneurial tubes, suggesting that DN-P1 could generate a better graft concerning the structural preservation and the 3D organization of the internal basal lamina of the nerve that could act as a proper structural support for the growing nerve, resembling results obtained by Hudson and colleagues [8].

TEM ultrastructural analysis showed that fragmented axonal debris were present in decellularized samples treated with DN-P1, while some intact axons were detected in DN-P2. These findings are very similar to data reported by previous studies using both the Hudson’s or Roosens’ protocols [30,32]. While Picrosirius red staining showed the collagen presence in both DN-P 1 and 2, ultrastructural TEM analysis showed that collagen fibers were present and well-conserved in the nerves decellularized with DN-P1, while in DN-P2, collagen fibers were not intact and severely disorganized. In TEM analysis, in both the employed protocols, the complete myelin elimination was not achieved, indeed, a disaggregated myelin structure was still detectable. This was in contrast with the results obtained by MCOLL histological staining that demonstrated the complete absence of myelin in DN-P1 treated nerves, as well for the Roosens’ protocol [30,32]. This could be attributed to the different sensitivity and tissue processing protocols of both techniques. For light microscopy, tissues are fixed in formaldehyde, dehydrated in ethanol, cleared in xylol, and then impregnated in warm paraffin. This aggressive process eliminates the tissue elements, such as myelin, which were altered and probably solubilized by the decellularization process. In contrast, sample preparation for TEM uses two stronger fixatives that stabilize proteins (glutaraldehyde) and lipids (osmium tetroxide), allowing the identification of elements that have resisted the decellularization process [23].

The differences seen in our study between the results obtained by light and electron microscopy are related to technical reasons. This demonstrates that the combination of immunohistochemistry, SEM, and TEM analyses is crucial to assess the rate of decellularization of a tissue. Histochemistry and immunohistochemistry provide an overview of the structure and distribution of some general or tissue-specific elements, while the ultrastructural analyses serve to confirm these findings, but more importantly to demonstrate accurately those elements that are not detected by conventional light microscopy, such as myelin or axonal remnants.

Both the decellularization protocols altered the nerve mechanical properties, in contrast with data reported by Roosens protocol [30,32], although in general, there is no consensus on the effects of decellularization detergents in affecting the mechanical properties of treated nerves. The Young’s modulus detected a lower viscoelastic properties compared to native nerves. This means that the decellularized nerves had a low resistance to deformation when a force is applied. This finding was coherent with the stress at fracture results requiring a lower force before the decellularized nerve rupture. The deformation level in nerves decellularized with DN-P1 was not significant with respect to native samples as measured by the strain at fracture, which was not the case of nerves treated with DN-P2 where a high deformation occurred at the point of fracture. These mechanical changes were expected, since most of the decellularization treatments slightly affect the ECM components, mainly collagen, which plays an important role in the nerve mechanical strength [21]. However, it has been shown that substitutes with biomechanical properties, not fully comparable to a native nerve, have been successfully used in experimental nerve injury repair [28,33], suggesting that our new allograft could be tested for future in vivo preclinical studies.

To evaluate the residual cytotoxicity of detergent remains, the viability of seeded rADMSCs onto the decellularized samples was detected. ADMSCs are widely used in various tissue engineering applications for their ease pf purification and access, in addition to their high in vitro proliferation capacity [12,34]. Moreover, in the view of cell reseeding for nerve grafts, the use of autologous MSCs drastically reduces the invasive collection of cells from nerves as well as the patient morbidity. Finally, the immunomodulatory properties of MSCs are exploited in cell therapy-resistant graft-versus-host disease, thus better controlling the autoimmunity and inflammatory responses [12].

DN-P1 treated nerves better support the cell viability compared to DN-P2, which decreased the cell viability and functionality. It could be related to the chemical agent used, which in the case of the DN-P2 decreased the cytocompatibility of the generated matrices. Perhaps some chemical agents within the acellular matrices or essential ECM molecules, which support the cell adhesion and function, were irreversibly eliminated, affecting the cell viability and functionality. It can be hypothesized that the use of PAA, barely employed in nerve decellularization [24,25], could have an impact on the nucleic acids, cellular remnants, and cytoplasm removal without altering the structure of ECM; it can be easily removed from the nerve following decellularization, thus being highly biocompatible and favoring the cell viability of newly seeded cells on decellularized nerves.

The combination of different detergents used in each decellularization protocol led to different results in the tested decellularized nerves. It is difficult to assess the exact effect of each detergent used alone, as the effects can be assessed at the end of the whole protocol including the subsequent use of different detergents. One main difference between protocols could be the combination of the physical forces, agitation in addition to repeated sonification cycles in DN-P2, that in our opinion could have accounted for the extensive endoneurial damage and the disruption of the collagen fibers, as detected in SEM and TEM analysis, respectively. The greater viability sustained by DN-P1 nerves could be attributed to a better removal of detergents residues as a result of the extensive washes alternating with distilled deionized water and PBS, while DN-P2 has less washes mainly in PBS. Another factor could be the harshness of the used detergents, Triton-X100 and SDS, compared to TBP and PAA, so even if complete detergent removal was not achieved, the toxicity would be less using the last-mentioned reagents. A detailed study solely focused on the use of different detergents and their exact possible adverse effects on the tissue could be helpful to choose the best combination of detergents for future decellularization protocols.

The small sample size (*n* = 3) could be considered a limitation of the present study; however, our proof of concept study was mainly focused on an overall evaluation of the outcomes of the decellularization protocol DN-P1 compared to DN-P2 already published for the nerves. Even though the sample size was small, it was enough to guide us to detect the second limitation of this study, which is the DNA content, as none of the tested protocols achieved the optimal DNA content (<50 ng/dry weight).

The overall results of the present in vitro study show that DN-P1 with minor modifications could be a more promising protocol than DN-P2 to decellularize nerves. DN-P1 is a decellularization protocol that uses a novel combination of two detergents, mainly TBP and PAA, that has been already efficiently used to decellularize tendons, but whose effect has never been tested on nerves before. Both DN-P1 and DN-P2 require short preparation time compared to others such as the Sondell protocol, but DN-P1 shows a better DNA component removal, ultrastructural organization, and cell viability compared to DN-P2. In order to confirm the effectiveness of DN-P1, further in vivo studies are necessary to detect if the leftovers from cellular remnants could provoke an adverse immune reaction and to assess the regeneration capability of decellularized nerves treated with DN-P1.

## 4. Materials and Methods

### 4.1. Sciatic Nerve Harvesting

A total number of 14 adult three-month-old female Wistar rats weighing 200–250 g (Envigo, Bresso, Milano, Italy) were euthanized in carbon dioxide chambers. Rats were placed on the dissecting board in dorsal recumbency to expose sciatic nerves. Briefly, a longitudinal incision was performed with a scalpel blade from the knees to the pelvis, exposing the underneath muscles. By blunt dissection, the gluteus maximus and biceps femoris muscles were split to expose the entire length of the sciatic nerve. Muscles were further cut horizontally reaching the spinal cord and vertically upward separating the external oblique muscle from the spinal cord. The sciatic nerves were isolated from surrounding connective tissues and cut starting from the lumbosacral region to their terminal branches, thus yielding a total length ranging from 25 to 30 mm [35]. The study was conducted according to the guidelines of the Animal Care and Use Committee (IACUC) of the University of Turin (Permit N. 864/2016-PR) approved on 14/9/2016.

### 4.2. Decellularization Protocols

Nerves were distributed into three main groups (*n* = 9 each), untreated nerves serving as controls while the other groups received two different decellularization treatments: DN-P1: 1% tri (n-butyl) phosphate (TBP) followed by 3% peracetic acid (PAA), as described in a successful decellularization protocol developed for tendons as previously published [11]; DN-P2: 125mM sulfobetaine-10 (SB-10), 0.2% TritonX-100 followed by 0.25% sodium dodecyl sulfate (SDS) as a decellularization protocol specifically described for nerves [22].

Following the decellularization treatments, nerves were cut into pieces 10 mm each and were randomly assigned for subsequent analyses (*n* = 3 for each analysis). To assess the mechanical properties, the whole nerve was used, ranging from 25 to 30 mm length (*n* = 3).

Briefly, in DN-P1, nerves were immediately dry-frozen at −80 °C until processing. Then, the specimens were thawed in phosphate buffer solution without sodium and magnesium (PBS-/-) at RT for 30 min. The decellularization started immersing nerves in 1% TBP in 1 M Tris-HCl pH 7.8 solution at RT for 24 h under agitation, then rinsing twice in ddH_2_O for 15 min. To remove detergents, nerves were moved to PBS-/- at 4 °C for 24 h, followed by a 4 h treatment with 0.0025% DNase-I at RT under agitation. After washing twice in ddH_2_O for 15 min and in PBS -/-, the nerves were incubated at RT in 3% PAA under agitation for 4 h. The final step consisted in double washes in ddH_2_O and PBS -/- for 15 min each.

In DN-P2, nerves were immediately incubated in PBS solution containing 125 mM SB-10, 0.2% TritonX-100, 1% penicillin/streptomycin under agitation for 48 h at RT, then dry-frozen at -80 °C until processing. After thawing, nerves were washed three times in PBS for 30 min and moved to 0.25% SDS in PBS solution under agitation for 30 min followed by 5 min sonication cycle at 40Hz. The last two steps were repeated five times, followed by three washes in PBS for 30 min. 

All decellularized nerves were stored at 4 °C in PBS supplemented with a ready-to-use commercially available antibiotic antimycotic solution (penicillin, streptomycin, and amphotericin B) until use. All reagents were bought from Sigma-Aldrich (Sigma Aldrich, Steinheim, Germany) unless otherwise specified.

### 4.3. Histology and Immunohistochemistry

For paraffin embedding, samples were fixed in 10% formalin solution for 48 h, and then dehydrated in ascending ethanol gradients, as standard. Briefly, the samples were treated for 1 h with 70% ethanol, then three 1 h passages in 95% ethanol, three 1 h passages in 99% ethanol, and one passage in Xylene for 30 min, followed by two more Xylene passages of 45 min each; finally, three passages in paraffin wax 60 °C, 1 h each. Then, nerve orientation was checked before the paraffin embedding; 5 µm sections were prepared. Hematoxylin and eosin (HE) staining was performed to assess the morphology and general structure of the nerves; proteoglycans and collagen were evaluated by Alcian blue and Picrosirius red staining, respectively. The evaluation of myelin and collagen reorganization pattern was performed using MCOLL method following a previously described protocol [36,37].

For immunohistochemical analyses, sections were incubated overnight at 4 °C with antibodies against basal membrane laminin (1:1000) (L8271, Sigma Aldrich, Steinheim, Germany), axonal neurofilaments (1:500) (approx.160 and 200 kDa_ NF-M/H_ intermediate filament) (N2912, Sigma Aldrich, Steinheim, Germany), vimentin (53kDa_ type III intermediate filament) for fibroblasts (1:200) (V6630, Sigma Aldrich, Steinheim, Germany), and S-100 (S100B, S100A1 and S100A6) protein for Schwann cells (1:400) (Z0311, DakoCytomation, Glostrup, Denmark), as previously described [23,30]. Negative controls (no primary antibody) were prepared to avoid non-specific staining. Secondary antibodies ImmPRESS IgG Rabbit (MP-7401, Vector Laboratories, Burlingame, EEUU) and Mouse (MP-7402Vector Laboratories Burlingame, EEUU) were used. For the specific nuclear DNA detection, 4′,6-diamidino-2-phenylindole (DAPI) fluorescent mounting was used and examined with a fluorescence microscope.

### 4.4. DNA Extraction and Quantification

DNA assay was performed to quantify the cellular remnants after the decellularization processes. DNA was extracted using the QIAamp^®^ mini kit (Qiagen, Hilden, Germany) following the manufacturer’s protocol. Briefly, nerve tissues were lysed by adding the provided kit ATL buffer and proteinase K at 56 °C for 3 h. Then, the second kit buffer AL was added, and samples were incubated at 70 °C for 10 min. After centrifuging, absolute ethanol was added. The whole mixture was then moved to QIAamp mini spin column and centrifuged at 6000× *g* for 1 min; the flow through and the collection tubes were discarded and the QIAamp mini spin column was placed in a new collection tube, and kit buffer AW1 was added. The tube was centrifuged at 600× *g* for 1 min, and flow-throw and collection tube was discarded. Kit buffer AW2 was added to the column and centrifuged at full speed for 3 min. As recommended by the manufacturer, the column was centrifuged for an extra minute at full speed to eliminate any buffer remaining. Finally, to elute DNA from the column, kit buffer AE was incubated at RT for 1 min followed by 6000× *g* centrifugation for 1 min. 

For each sample, DNA concentration was detected by a NanoDrop 2000 spectrophotometer (Thermo Fisher Scientific, Waltham, MA, USA), five measures were taken for each tested sample, and the measurements were then normalized to the original dry weight of the samples before extraction.

### 4.5. Ultrastructural Analyses 

For scanning electron microscope (SEM), samples were fixed in 2.5% glutaraldehyde 0.05 M cacodylate buffer (pH 7.2) for 6 h at 4 °C, followed by three washes in cacodylate buffer at 4 °C, then fixed in osmium tetroxide for 1 h at 4 °C and dehydrated in increasing acetone concentrations of 30%, 50%, 70%, and 90% for 15 min each at 4 °C. The last two concentrations of 96% and 100% acetone lasted 25 min each, and samples were subjected to critical point drying (CPD), where acetone present in the samples is exchanged with liquid carbon dioxide (CO_2_), which undergoes a phase transition to gas in a pressurized chamber. Finally, the samples were mounted on SEM specimen stubs by means of carbon based electrically conductive double-sided adhesive discs, and sputter coated with gold. Images were acquired using FEI Quanta 200 environmental scanning electron microscope (FEI Europe, Eindhoven, Netherlands) [30].

For transmission electron microscope (TEM), samples were embedded in resin. Specifically, samples were fixed in 2.5% glutaraldehyde followed by 2 h post fixation in 2% osmium tetroxide; later samples were dehydrated by fully immersing in ascending ethanol gradients (30%, 50%, 70%, 80%, 95%, and 100%). As ethanol is immiscible with resin and to be totally removed, samples were treated twice in a transitional solvent of propylene oxide for 7 min. Finally, samples were left 1 h in a pre-infiltration solution of equal parts of propylene oxide and Glauerts resin mixture (Araldite M and Araldite Harter, HY 964 in the ratio 1:1, 0.5% dibutyl phthalate plasticizer; plasticizer is added to reduce resin viscosity and hence allow better embedding medium infiltration into the specimen, and it also improves the final block sectioning) and overnight in the Glauerts resin mixture. Then, 2% accelerator DMP-30 was used to promote the polymerization of the resin embedding medium, and samples were incubated in oven at 60 °C for 2–3 days. Later, 2.5 mm semi-thin sections and 70 nm ultrathin sections were prepared using an Ultracut UCT ultramicrotome (Leica Microsystems, Wetzlar, Germany). Semi-thin sections were attached to normal glass slide and stained with 1% toluidine blue for high resolution light microscopy examination using a DM4000B microscope equipped with a DFC320 digital camera and an IM50 image manager system (Leica Microsystems, Wetzlar, Germany). Ultrathin sections were collected on a pioloform coated grid. The day after, specimens were counterstained using a solution of uranyl acetate replacement (Electron Microscopy Sciences Hatfield, PA, USA) and analyzed by means of a JEM-1010 transmission electron microscope (JEOL, Tokyo, Japan) equipped with a Megaview-III digital camera and a Soft-Imaging-System (SIS, Münster, Germany). Images were acquired for different sections at different magnifications [38]. 

### 4.6. Biomechanical Characterization

Biomechanical responses of decellularized nerves were assessed to determine the effect of the decellularization process on their elastic and tensile properties, as previously described [30,39]. The test was performed using an electromechanical testing apparatus Instron 5943 (Instron, Needham, MA, USA) using the software Bluehill 3.62 with a 50 N charge cell load. Nerves were clamped at 10 mm distance; a tensile uniaxial stress was applied to the nerves using pre-set parameters of constant strain rate 10 mm/min. Tensile strength was applied to the nerve until its failure was achieved. Young’s modulus (MPa), stress of fracture (MPa), strain of fracture (%), and extension at fracture (mm) were measured. 

### 4.7. Cell–Biomaterials Interactions Analyses

To evaluate the cytotoxicity of the decellularized nerves, as a consequence of the decellularization procedures (residual detergents), rat adipose-derived mesenchymal stem cells (rADMSCs) were used. Cells were seeded on the inner surface of decellularized nerves, and their morphology, viability, and cellular metabolic activity were determined using the LIVE/DEAD^TM^ Viability/Cytotoxicity Kit (L/D) (# L3224, Thermo-Fisher Scientific, Portland, OR, USA) [30] and WST-1 assay (Roche, Mannheim, Germany), respectively, as previously described [40].

For these analyses, 24 multi well plates were pre-coated with agarose type I to target the cell adherence and growth onto the decellularized nerves and prevent their possible adherence to the well bottom. A longitudinal incision was made along the whole nerve length and was opened and placed flatly on the pre-prepared agarose surface. Then, 2 x10^4^ rADMSCs (passage 7) were seeded on the inner nerve surface of each decellularized nerve, then cultured in complete Dulbecco’s modified Eagle’s medium (DMEM, 10% FBS, 1% of commercial ready Antibiotic Antimycotic mix, all from Sigma-Aldrich #D6429, F7524, and A5955, respectively (Sigma-Aldrich, Steinheim, Germany)) and incubated under normal culture conditions (37 °C, 5% CO_2_, 95% humidity). After 48 h of incubation, the medium was removed, and cells were rinsed once with PBS. 

L/D assay was conducted according to manufacturer’s instructions. In this sense, 300 µL of calcein AM and ethidium homodimer-1 mixture was added to each cell culture and incubated for 15 min. Finally, cells were observed using Nikon Eclipse Ti fluorescence equipped with a Nikon DXM 1200c Digital Camera (Nikon, Tokyo, Japan). Viable and metabolically active cells incorporated the supravital fluorochrome calcein and appeared green, while dead cells, with irreversible cell membrane damage, incorporated the ethidium fluorochrome and emitted a red nuclear fluorescence.

For WST-1 (water-soluble tetrazolium salt-1), a colorimetric biochemical assay was conducted. This method allows the quantification of the activity of the mitochondrial dehydrogenase, which cleavages tetrazolium salt into formazan, demonstrating the presence of viable and metabolically active cells. The greater the number of viable cells, the greater the amount of the formazan produced. Following manufacturer instructions, seeded cells on nerves were incubated in 450 µL of WST-1 working solution for 4 h under standard culture conditions. In a 96 well plate, the total incubated solution for each nerve (*n* = 3) was distributed into four wells of 100 µL each (four readings/each nerve). Absorbance was measured at 450 nm by means of ASYS UVM340 spectrophotometer using the DigiRead software (Biocrom Ltd., Cambridge, UK).

In both methods 2 x10^4^ rADMSCs were seeded in non-coated wells as 2D positive or negative technical controls. To generate 2D negative control, an irreversible cell-membrane and nuclei damage was induced using 2% Triton X-100. WST-1 obtained values from both tested groups; DN-P1 and DN-P2 were normalized to 2D positive control where it represents 100% viability [32].

### 4.8. Semi-Quantitative Histological and Immunohistochemical Analysis

Semi-quantitative analyses were performed on histochemically or immunohistochemically stained sections. In this sense, ImageJ software (National Institute of Health, Bethesda, MD, USA) was used to select the adequate color threshold. Once positive reactions were isolated, the percentage of area occupied by each histochemical or immunohistochemical reaction (or area fraction) was measured as previously described [4]. For each positive reaction, color threshold selection was chosen using the adequate Hue value, and the saturation and intensity were optimized for native samples. The same parameters were then followed for the decellularized samples. The percentage of positive stained area was calculated in function of the entire nerve area at 10x. For these analyses, three independent nerve samples were measured per each staining for natives and decellularized nerves.

### 4.9. Statistical Analysis 

Statistical analyses were conducted using SPSS (SPSS Inc., Chicago, IL, USA). Comparison between two different independent groups were analyzed using the nonparametric Mann–Whitney U test. Analyses were performed on quantitative and semiquantitative data. In this study, *p* < 0.05 were considered statistically significant for all two-tailed tests.

## Data Availability

All data are available upon request from authors.
